# Evaluation of Ethanol Coproducts as Sustainable Protein Sources in Pacific White Shrimp (*Litopenaeus vannamei*) Diets

**DOI:** 10.1155/anu/9151629

**Published:** 2025-09-25

**Authors:** Trinh H. V. Ngo, Timothy J. Bruce, Julio C. García, Luke A. Roy, D. Allen Davis

**Affiliations:** ^1^School of Fisheries, Aquaculture and Aquatic Sciences, Auburn University, Auburn, Alabama, USA; ^2^USDA-ARS, Aquatic Animal Health Research Unit, Auburn, Alabama, USA

**Keywords:** corn-fermented protein, gene expression, growth performance, Pacific white shrimp *L. vannamei*

## Abstract

Use of corn-fermented protein (CFP), a new product produced using Fluid Quip Technologies, as a protein source in aquaculture feeds, constitutes a promising option due to its dependable supply and cost-effectiveness. In this study, two growth trials were performed to evaluate the effectiveness of CFP products such as CFPA (48% crude protein [CP]), CFPB1 (50% CP), and CFPB2 (60% CP) from two different sources (A and B) in practical diets for juvenile Pacific white shrimp (*Litopenaeus vannamei*). The test diets in both trials were formulated to be isonitrogenous and isolipidic (36% crude protein and 6% crude lipid). These diets were produced by supplementing the basal diet with 5%, 10%, 15%, and 20% CFPA or 4%, 8%, 12%, and 16% of CFPB1 and CFPB2 to replace soybean meal (SBM) on an isonitrogenous basis. In a 6-week experiment, shrimp (1.02 ± 0.02 g mean weight, 15 shrimp per tank, *n* = 6) were offered CFPA diets. Growth parameters and protein retention showed no significant differences among diets. However, a significant feed conversion ratio (FCR) increase was observed when shrimp were fed a diet containing 20% CFPA compared to the basal and 5% CFPA diets. Physiological gene expression analysis revealed no signs of gut inflammation or shifts in hepatopancreas digestive enzymes (*p*  > 0.05). However, the expression of immune-related *tnf-α* gene was significantly upregulated (*p*  < 0.05) in shrimp-fed CFPA 25% diet compared to CFPA 5% and control groups. In a 7-week experiment, shrimp (0.55 ± 0.01 g, 15 shrimp per tank, *n* = 5) received experimental CFPB1 or CFPB2 diets. No differences were observed in growth performance. This research highlights the potential of CFP as a protein source in shrimp diets by expanding the range of feed ingredients and identifying optimal inclusion levels.

## 1. Introduction

The increasing global demand for shrimp has encouraged the aquaculture industry to adopt more sustainable and efficient feeding methods. Traditionally, shrimp diets have predominantly depended on fish meal (FM) as a protein source due to its amino acid (AA) composition and high digestibility [1]. However, relying on FM creates significant challenges. The expanding demand for this limited resource, combined with fluctuating prices and serious environmental concerns, such as overfishing and ecological damage, has driven an exploration for more sustainable protein source alternatives [2]. This transition corresponds with the broader objective of reducing the aquaculture industry's dependance on limited marine resources and fostering more sustainable practices. The search for alternative protein sources has resulted in an expansion of plant-based proteins, with soybean meal (SBM) becoming the primary alternative [3–5]. SBM offers a dependable supply and lower cost compared to FM and other animal proteins, which has uniquely positioned it as an appealing choice for aquaculture nutrition [6, 7]. While widely adopted, SBM does present certain nutritional challenges. Factors such as antinutritional compounds and reduced digestibility in crustaceans can influence their effectiveness and potentially negatively impact growth performance [8, 9]. Furthermore, heightened awareness of the environmental ramifications of SBM production, including deforestation, changes in land use, and greenhouse gas emissions, has prompted inquiries into its long-term viability. These issues underscore the pressing necessity for more circular and environmentally sustainable protein sources to advance the future of aquaculture.

Corn-fermented protein (CFP), a new coproduct of ethanol production, is recognized as an innovative protein source for the nutrition of aquatic animals [10]. It comes from the bioethanol production process and facilitates a circular bioeconomy by converting agricultural byproducts into high-value, protein-rich feed ingredients. Each year, the United States produces approximately 15.4 billion gallons of ethanol, yielding around 36.4 million metric tons of coproducts, including distillers' dried grains with solubles (DDGS), corn gluten meal (CGM), and CFP, according to Langholtz et al. [11]. CFP stands out due to its high protein content, low fiber, and enhanced digestibility, which is a product of the fermentation process [10]. Its nutritional benefits may also be attributed to the presence of residual *Saccharomyces cerevisiae*, a yeast recognized as a valuable source of essential AAs (EAAs) and bioactive compounds that support growth and health in aquatic species [12, 13]. Produced through advanced fractionation technologies, such as the maximized stillage coproducts system (Fluid Quip Technologies, Cedar Rapids, Iowa, USA), CFP is obtained by mechanically separating nonprotein components from whole stillage, resulting in coproducts with crude protein levels approaching 50%, which exceeds that of SBM [14]. This process enhances nutrient bioavailability and reduces antinutritional factors, addressing some of the challenges commonly associated with plant-based protein sources, such as SBM [15]. CFP offers a cost-effective and consistently available alternative to SBM while also supporting the principles of a circular bioeconomy. Ethanol coproducts have shown potential in the diets of species such as Nile tilapia (*Oreochromis niloticus*), European seabass (*Dicentrarchus labrax*), Atlantic salmon (*Salmo salar*), and channel catfish (*Ictalurus punctatus*) [16–19], though their application in shrimp nutrition remains underexplored. Considering the pressing demand for sustainable protein sources that align with circular bioeconomy concepts, evaluating CFP in shrimp diets is a crucial step in developing more environmentally responsible feed formulations.

This study investigates the potential of CFP sources as sustainable alternatives to SBM in the diets of *Litopenaeus vannamei*, with a focus on growth performance, feed efficiency, and the expression of genes linked to digestive and immune functions under clear water conditions.

## 2. Materials and Methods

### 2.1. Experimental Diets

All experimental diets in both trials were formulated to be isonitrogenous and isolipidic (36% crude protein and 6% crude lipid) and prepared at the Aquatic Animal Nutrition Laboratory at the School of Fisheries, Aquaculture, and Aquatic Sciences, Auburn University (Auburn, Alabama, USA) using standard procedures for shrimp feeds [20]. Proximate and AA compositions for each of the main ingredients are shown in Table 1. In both trials, the basal diet mainly consisted of FM, poultry meal (PM), SBM, and corn protein concentrate as primary protein sources, along with fish oil and soybean oil as lipid sources. In Trial 1, five experimental diets were formulated by progressively replacing SBM (51.69% included in diets) with 0%, 10%, 15%, 20%, and 25% of CFPA. In Trial 2, six experimental diets were developed to include increasing five levels of CFPB1 (0%, 4%, 8%, 12%, and 16%) and four levels of CFPB2 (0%, 8%, 12%, and 16%) as partial substitutes for SBM (42.6% included in diets). Briefly, diets were prepared by mixing the preground dry ingredients and oil in a food mixer (Hobart, Troy, Ohio, USA) for ten to fifteen minutes. Boiling water (~30%–40% by weight) was mixed into the ingredients to achieve a consistency suitable for pelleting. The mixture was then processed into pellets using a meat grinder fitted with a 3 mm die. The resulting wet pellets were dried overnight in a fan-ventilated oven at temperatures below 35°C to reduce the moisture content to 8%–10%. A portion of each diet was crumbled and sieved to the desired size for use during the initial stages of the study when the shrimp were too small to consume 3 mm pellets. All diets were packed and stored in a freezer at –20°C until needed for the experiment. The proximate composition (Tables 2 and 3) and AA profiles (Tables 4 and 5) of the experimental diets were analyzed at the University of Missouri Agricultural Experimental Station Chemical Laboratories (Columbia, Missouri, USA).

### 2.2. Growth Trial

Post larvae (PLs) of Pacific white shrimp (~0.005 g) were obtained from Homegrown Shrimp Hatchery (Indiantown, Florida, USA) and nursed in an indoor recirculating system and acclimated for several weeks. During this period, PLs were fed a commercial diet, PL Raceway Plus (Zeigler Bros. Inc., Gardners, Pennsylvania, USA), four times daily to an appropriate size (<0.1 g). All trials were conducted in a semiclosed recirculation system at E.W. Shell Fisheries Center at the Auburn University (Auburn, Alabama, USA).

The recirculating aquaculture system used for the study consisted of 36 and 40 tanks for Trials 1 and 2, respectively, all of which were connected to a shared 800 L reservoir tank equipped with a biological filter, bead filter, recirculating water pump, and supplemental aeration. For the growth trial, shrimp PLs of uniform size were manually selected and stocked at a density of 15 individuals per aquarium (120 L; internal dimensions: 0.52 m × 0.52 m × 0.50 m). Each dietary treatment was randomly assigned to six replicate groups in Trial 1 (initial mean weight 1.02 ± 0.02 g) and five replicate groups in Trial 2 (initial mean weight 0.55 ± 0.012 g). Shrimp were hand-fed the experimental diets four times daily at approximately 0800, 1100, 1300, and 1600 h. Weekly shrimp counts were conducted to adjust daily feed inputs, with rations calculated based on expected growth, assuming a feed conversion ratio (FCR) of 1.6. Shrimp were counted once a week to adjust daily feed input.

At the conclusion of the 6-week (Trial 1) and 7-week (Trial 2) growth trials, shrimp were collectively counted and weighed by replicate tank. Key performance metrics, including average final weight (FW), weight gain (WG), percentage WG (PWG), thermal-unit growth coefficient (TGC), survival (%), FCR, apparent net protein retention (ANPR), and weekly WG (WWG), were calculated at the end of growth trials. In Trial 1, samples of shrimp from the initial population, along with four shrimp per tank, were randomly selected, bagged, and stored at −20°C for later proximate analysis. Three additional shrimp were dissected to collect hepatopancreas and abdominal intestine tissues (segments 1^st^–5^th^), which were preserved in 800 and 300 µL of RNA Shield (Zymo Research, Irvine, California, USA), respectively, in 5 mL containers and 1.5 mL microcentrifuge tubes for further gene expression analysis. The frozen shrimp samples were thawed, dried in an oven at 95°C for 24 h, ground, and sent to Midwest Laboratories Inc. (Omaha, Nebraska, USA) for whole-body analysis. The results were used to determine the following feed efficiency parameters:

  $$\begin{array}{c} \text{Survival }\left(\%\right)\;=\text{Number of shrimp at termination}/\text{number of shrimp at the start }\times \;100, \end{array}$$

  $$\begin{array}{c} \text{Final weight }\left(\text{FW},g\right)\;=\text{Total biomass }\left(g\right)/\text{number of shrimp at termination}, \end{array}$$

  $$\begin{array}{c} \text{Weight gain }\left(\text{WG},g\right)\;=\text{Final weight }\left(g\right)\;-\text{initial weight }\left(g\right), \end{array}$$

  $$\begin{array}{c} \text{Percentage weight gain }\left(\text{PWG},\text{ \%}\right)\;=\text{Weight gain }\left(g\right)/\text{initial weight }\left(g\right)\times 100, \end{array}$$

  $$\begin{array}{c} \text{Weekly weight gain }\left(\text{WWG},\text{ g}\right)=\text{Weight gain }\left(g\right)/\text{culture period }\left(\text{weeks}\right), \end{array}$$

  $$\begin{array}{c} \text{TGC }=\;\left({\text{Final weight}}^{1/3}-{\text{ initial weight}}^{1/3}\right)/\left(\text{water temperature }\left({}^{\circ}C\right)\;\times \text{days}\right)\times 100, \end{array}$$

  $$\begin{array}{c} \text{Feed conversion ratio }\left(\text{FCR}\right)=\text{Total feed consumed }\left(g\right)/\text{weight gain }\left(g\right)\times 100, \end{array}$$

  $$\begin{array}{c} \text{ANPR }\left(\%\right)=\;\left[\text{FW }\left(g\right)\times \text{final protein content }\left(\%\right)\right]-\;\left[\text{IW }\left(g\right)\times \text{initial protein content }\left(\%\right)\right]/\text{protein offered }\left(g\right)\;\times 100. \end{array}$$

### 2.3. Water Quality

Dissolved oxygen (DO) levels were maintained close to saturation in each culture tank using air stones, which were connected to a shared airline powered by a regenerative blower (Model R4P115, Gast Manufacturing, Benton Harbor, Michigan, USA). Saltwater was prepared by mixing manufactured sea salt (Crystal Sea Marinemix, Baltimore, Maryland, USA) with dechlorinated freshwater and maintained at around 9.49–9.78 ± 0.2 ppt during the trials. DO, salinity, and water temperature were monitored twice daily with a YSI-2030 pro digital oxygen/temperature meter (YSI Corporation, Yellow Springs, Ohio, USA). Total ammonia nitrogen (TAN) and nitrite nitrogen levels were measured twice weekly using a YSI-9300 photometer (YSI, Yellow Springs, Ohio, USA). Water pH was checked twice per week throughout the experimental period using a pHep meter (Hanna Instrument, Smithfield, Rhode Island, USA).

### 2.4. Gene Expression Analysis

The intestinal and hepatopancreatic samples were homogenized in a 96-deep well plate containing 0.1 mm ceramic beads using a Bead Ruptor 96 (Omni International, Kennesaw, Georgia, USA) at 30 Hz for 20 min (5 min homogenization, 5 min rest) following the manufacturer's protocol. A total of 100 µL of homogenized RNA samples was extracted and purified using the MagMAX mirVana Total RNA Isolation Kit (Thermo Fisher Scientific, Waltham, Massachusetts, USA). Sample concentration and quality were measured using a BioTek Take3 Trio spectrophotometer (Agilent, Santa Clara, California, USA), ensuring A260:A280 ratios greater than 1.8. The extracted RNA was diluted and standardized to 40 ng µL^−1^ (intestine) and 30 ng µL^−1^ (hepatopancreas) before cDNA synthesis using the High-Capacity cDNA Reverse Transcription Kit (Applied Biosystems, Waltham, Massachusetts, USA). The experiment utilized four target genes: *tnf-α*, *tgf-β1*, Prophenoloxidase (*propo*), and *sod*; *tryp*sin, alkaline phosphatase (*alp*), *amylase*, and fatty acid synthase (*fasn*) (for intestine and hepatopancreas, respectively), with two reference genes (*ef1α* and *l21*) (Table 6). Primer efficiency (90%–110%) was validated using five serial dilutions (dilution factor of 2). Reference gene stability was assessed using NormFinder [28], identifying *ef1α* and *l21* as the most stable, with the lowest stability scores of 0.04 and 0.11 for the intestine and hepatopancreas, respectively. Primer efficiency was calculated as follows:

  
$$\begin{array}{c} \text{Efficiency }\left(\%\right)=10^{\left[\left(-1\right)/\text{Slope}\right]}\;-1\times \;100. \end{array}$$

qPCR was performed on a Bio-Rad CFX Opus 384 system using SsoAdvanced Universal SYBR Green Supermix (Bio-Rad, Hercules, California, USA). Each reaction (10 µL) contained 6 µL of master mix, 0.25 µL of each primer (stock concentration of 10 µM), 0.5 µL of nuclease-free water, and 4 µL of diluted cDNA. The thermal cycling profile consisted of 50°C for 2 min, followed by 95°C for 2 min, and then 40 cycles of 95°C for 15 s, 58°C for 15 s, and finally 72°C for 30 s. Relative gene expression was determined using the 2^*−*ΔΔCt^ method [29], where target gene expression was normalized to the geometric mean of the reference genes and compared to the basal treatment (set as expression = 1).

### 2.5. Statistical Analysis

All data were processed and analyzed using SAS (Version 9.4, SAS Institute, Cary, North Carolina, USA). Growth data from Trial 1 were evaluated using one-way ANOVA, while Trial 2 data were analyzed using ANCOVA to compare CFPB1 and CFPB2. In cases where significant differences were detected, Tukey's test was applied to distinguish variations among the experimental treatments. Additionally, linear, quadratic, and broken-line regression models were assessed using the coefficient of determination, corrected Akaike information criterion (AICc), weighted AICc, relative likelihood, and evidence ratio [30]. The coefficient of determination and *p*-value of the best-fit models were reported, along with results from a one-way ANOVA. The relationships between the inclusion levels of CFP and FW, WG, ANPR, and FCR were plotted and visualized using R4.3.3 [31] and RStudio 2023.12.1.402 [32], with 95% confidence intervals. A significance level of *α* = 0.05 was used for all statistical analyses.

## 3. Results

### 3.1. Water Quality

All water quality parameters in both trials were kept within the optimal range necessary for the growth and development of Pacific white shrimp, as recommended by Boyd and Tucker [33]. As shown in Table 7, the mean ± standard deviation for total ammonia, nitrite, and DO were 0.08 ± 0.05 mg L^−1^, 0.14 ± 0.14 mg L^−1^, and 7.23 ± 0.32 mg L^−1^, respectively, in Trial 1 and 0.12 ± 0.07 mg L^−1^, 0.23 ± 0.27 mg L^−1^, and 6.95 ± 0.27 mg L^−1^, respectively, in Trial 2.

### 3.2. Growth Performance

#### 3.2.1. Trial 1

**Figure 1:**
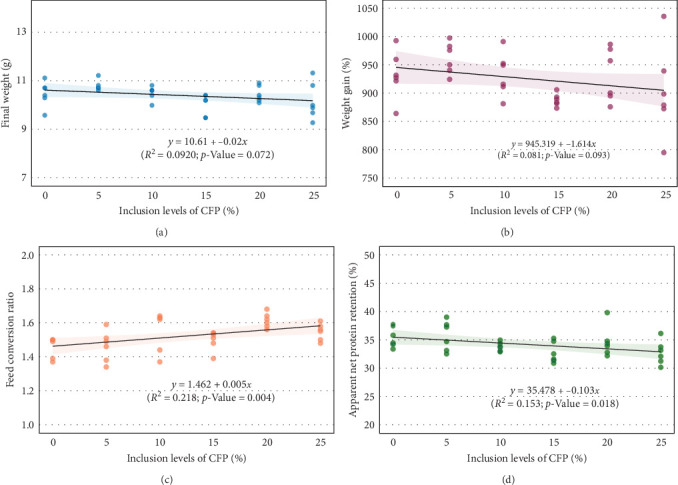
Relationship between various levels of corn-fermented protein (CFPA) and growth performance, including final weight (A), percentage weight gain (B), feed conversion ratio (C), and apparent net protein retention (D) of Pacific white shrimp (*Litopenaeus vannamei*)-fed experimental diets during 6 weeks (*n* = 6). CFP, corn-fermented protein A (Altipro).

Growth performance, feed utilization, and biometric parameters of *L. vannamei* fed various experimental diets over a 6-week period were presented in Table 8. Results showed that shrimp-fed the CFPA 5% diet achieved the highest FW (10.78 g), WG (9.77 g), and thermal growth coefficient (0.28), though these differences were not statistically significant (*p*  > 0.05). Furthermore, FCR was significantly influenced by CFPA levels (*p* = 0.007), with the lowest FCR (1.46) observed in the basal and CFPA 5% groups, indicating better feed efficiency, while the CFPA 20% group showed the highest FCR (1.61). Survival ranged from 82% to 93% across treatments, with no significant differences (*p* = 0.140). ANPR was highest in the CFPA 5% group (35.72%) and lowest in the CFPA 15% and 25% groups (32.73% and 32.75%, respectively), though these differences were not statistically significant (*p* = 0.062). Additionally, regression analysis revealed weak relationships between CFPA levels and most performance metrics, except for FCR (*R*^2^ = 0.218, *p* = 0.004) and ANPR (*R*^2^ = 0.153, *p* = 0.018), which showed significant trends (Figure 1C,D, respectively).

The whole-body proximate composition did not show noticeable differences across crude protein, crude fat, fiber, moisture, and dry matter (Table 9). Moisture (76.78%–77.77%) and dry matter (22.23%–23.22%) were consistent across treatments (*p*  > 0.05). Crude protein (76.03%–76.97%), crude fat (5.80%–6.85%), and fiber (5.56%–7.95%) also showed no significant variations (*p*  > 0.05). In contrast, ash content demonstrated a significant increase with higher CFPA inclusion levels (*p* = 0.05), and ranged from 11.07% in the CFPA 5% group to 11.95% in the CFPA 25% group. Regression analysis revealed a weak but significant relationship between CFPA levels and ash content (*R*^2^ = 0.177, *p* = 0.011), while no significant trends were observed for other proximate components.

**Figure 2:**
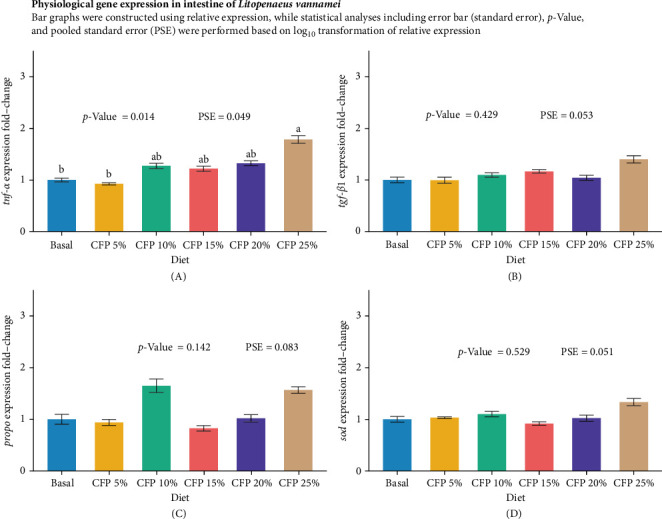
Quantitative real-time RT-PCR analysis of immune-related genes in 1^st^– 5^th^ intestine of Pacific white shrimp (*Litopenaeus vannamei*) cultured in a clear water system for 6 weeks (*n* = 6). Each replicate consisted of two technical replicates in one qPCR reaction. Figures (A–D): expression of *tnf-α* (A), *tgf-β1*(B), *propo* (C), and *sod* (D). Values not sharing a common superscript are significantly different (*p*  < 0.05). CFP, corn-fermented protein A (Altipro); PSE, pool standard error.

**Figure 3:**
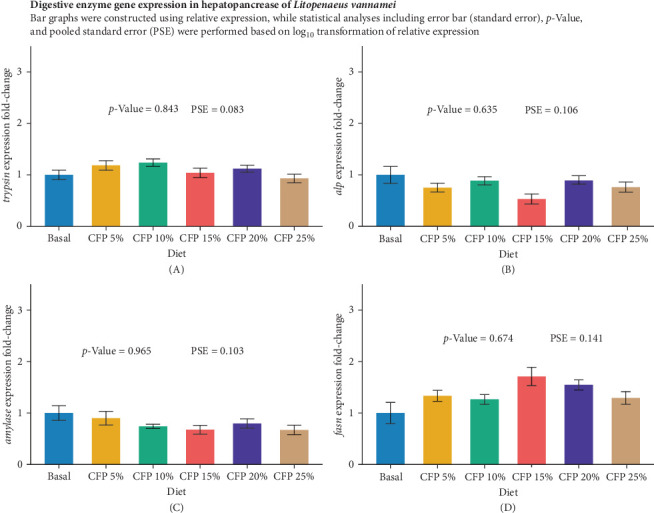
Quantitative real-time RT-PCR analysis of immune-related genes in hepatopancreas of Pacific white shrimp (*Litopenaeus vannamei*) cultured in a clear water system for 6 weeks (*n* = 6). Each replicate consisted of two technical replicates in one qPCR reaction. Figures (A–D): expression of *tryp*sin (A), *alp* (B), *amylase* (C), and *fasn* (D). Values not sharing a common superscript are significantly different (*p* < 0.05). CFP, corn-fermented protein A (Altipro); PSE, pool standard error.

Gene expression analysis in the intestine showed similar expression across diets, with no statistically significant upregulation or downregulation observed for *tgf-β1*, *propo*, and *sod* genes (*p*  > 0.05; Figure 2). The proinflammatory cytokine *tnf-α* showed a significant upregulation in shrimp-fed higher inclusion levels of CFPA (*p* = 0.014), with the highest expression observed in the CFPA 25% diet, with a fold change of ~1.52 compared to the basal diet (Figure 2A). Shrimp fed the basal and CFPA 5% diets exhibited the lowest *tnf-α* expression, while those fed CFPA 10%, 15% and 20% diets showed intermediate values without significant differences among them (*p*  > 0.05). In contrast, the transforming growth factor β1 (*tgf-β1*) presented a gradually increasing trend as CFPA inclusion increased, although differences were not statistically significant (*p* = 0.429, Figure 2B). Immune-related genes, *propo*, and sod genes were not significantly affected by dietary treatments (*p*  > 0.05). However, shrimp fed CFPA 10% and 25% diets tended to show higher *propo* expression compared to the basal diet (Figure 2C,D). In the hepatopancreas, gene expression analysis of digestive enzyme genes was not significantly influenced by dietary treatments (*p*  > 0.05; Figure 3). The expression of trypsin (*p* = 0.843) peaked at approximately 1.24 fold-change in the CFPA 10% diet but declined to approximately 0.93 fold-change in the CFPA 25% diet (Figure 3A). Similarly, *alp* and *amylase* showed consistent downregulation across CFPA diets (*p*  > 0.05), with the lowest expressions observed at CFPA 15% and CFPA 25% groups, respectively (Figure 3B,C). In contrast, the lipid metabolism gene *fasn* exhibited a trend of increasing expression with higher CFPA inclusion, reaching a peak of approximately 1.71 fold-change in the CFPA 15% diet, although this effect was not statistically significant (*p* = 0.674, Figure 3D).

#### 3.2.2. Trial 2

**Figure 4:**
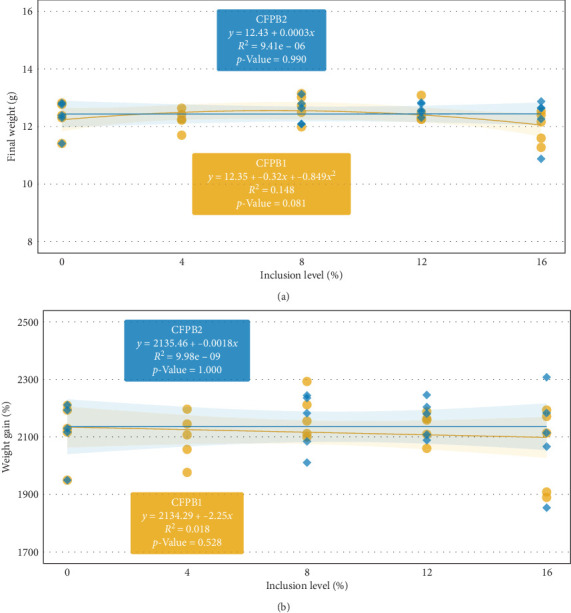
Relationship between various levels of corn-fermented protein B (CFPB1/2) and growth performance, including final weight (A) and percentage weight gain (B) of Pacific white shrimp (*Litopenaeus vannamei*)-fed experimental diets during 7 weeks (*n* = 5). CFPB, Ultra high protein products (50% and 60% crude protein), Green Plains, Omaha, Nebraska, USA.

The growth performance of juvenile Pacific white shrimp fed diets containing graded levels of corn-fermented products (CFPB1 and CFPB2) over 7 weeks was presented in Table 10. FW, WG, PWG, TGC, FCR, survival, and WWG were not significantly influenced by dietary treatments (*p*  > 0.05). Shrimp-fed CFPB1 at 8% inclusion exhibited the highest FW (12.67 g) and WG (12.12 g), while those fed CFPB1 16% had the lowest values (11.99 and 11.43 g, respectively). Similarly, the CFPB2 12% group yielded slightly higher growth parameters than the basal and the other CFPB2 treatments, but the differences were not statistically significant. FCR ranged from 1.38 to 1.52 across all treatments and showed no clear trend as CFP inclusion increased. Survival remained high (84%–91%) and did not differ among diets. The ANCOVA results indicated no significant interaction between ingredient type (CFPB1 and CFPB2) and inclusion levels for any of the growth parameters examined (*p*  > 0.05). Based on regression analysis (Figure 4) for CFPB1, the quadratic model provided the best fit for the FW (*R*^2^ = 0.148, AIC = 38.492) and indicated a nonlinear relationship, while the linear model was more suitable for WG percentage (*R*^2^ = 0.018, AIC = 305.331). In contrast, for CFPB2, the linear model was the most appropriate for both FW (*R*^2^ = 9.41e-06, AIC = 36.826) and WG percentage (*R*^2^ = 9.98e-09, AIC = 249.967), suggesting a minimal influence of CFPB2 inclusion levels on these growth metrics.

## 4. Discussion

**Figure 5:**
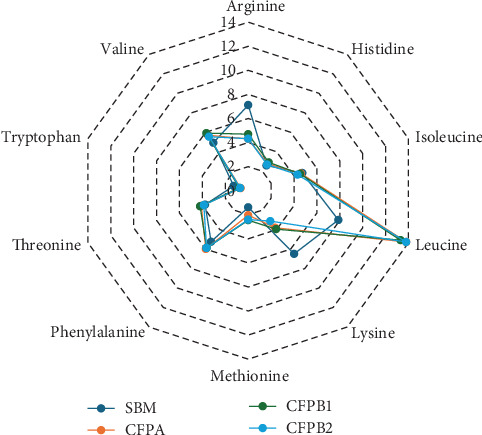
Spider graph comparing the essential amino acid profiles of soybean meal and corn-fermented products (CFPA and CFPB) as a percentage of total protein. CFPA, Altipro product, Renewable Products Marketing Group, Shakopee, Minnesota, USA; CFPB, Ultra high protein products (50% and 60% crude protein), Green Plains, Omaha, Nebraska, USA.

This study evaluated the performance of juvenile Pacific white shrimp offered two new sources of CFP (CFPA and CFPB1/2), produced through innovative processing technology in the dry grind ethanol industry. The AA profiles and proximate composition of CFP products and SBM were different and highlighted notable differences between these two plant-based protein sources in shrimp diets (Table 1 and Figure 5). Compared to SBM, CFP products contained higher levels of crude protein (48.6%–61.16% compared to 43.28% for SBM) and crude fat (3.61%–4.79% against 0.26% for SBM), but lower levels of lysine, which is the first limiting AA in shrimp diets because of its essential function in growth promotion, feed efficiency, and stress resistance [34]. However, CFP had higher levels of methionine and leucine, with methionine playing a crucial role in immune function and the regulation of oxidative stress [35]. These variations suggest that CFP may function as a more concentrated protein source, and potentially improve protein retention in shrimp diets, provided that lysine deficiencies are properly managed [36]. While the digestibility of corn-derived proteins may be lower than that of SBM due to fibrous components and non-starch polysaccharides [37], the increased methionine and lipid content may enhance sulfur AA balance and energy availability.

In Trial 1, results demonstrated that the inclusion of CFPA in diets for juvenile Pacific white shrimp did not significantly influence growth performance, although notable trends were observed. The significant FCR improvement at 5% CFPA inclusion, followed by a decline in ANPR at higher levels, suggests an optimal threshold for this ingredient in juvenile shrimp diets. These findings are consistent with the research of Qiu [38], which demonstrated that high protein distiller's dried grain (HPDDG), which uses a different processing technology, can be incorporated up to 30% in shrimp diets as a substitute for SBM without negatively affecting growth performance. Guo [12] reported that a diet comprising 20% HPDDG for Pacific white shrimp did not adversely affect growth performance under green water conditions over a 56-day feeding period. HPDDG has the advantage of a higher protein content compared to traditional DDGS, thereby fulfilling shrimp's dietary protein needs at reduced inclusion levels, thus allowing greater flexibility in diet composition [39]. The results of this study suggest that improved feed efficiency at lower CFPA inclusion levels may result from improved nutrient digestibility through fermentation, which reduces antinutritional factors and increases the bioavailability of EAAs.

Previous studies on alternative protein sources in aquatic animal diets have shown varied effects on whole-body composition, but our findings provide clearer insight. For example, Hu et al. [40] and Tian et al. [41] found that while dietary protein can increase body protein content, it can lead to inconsistent changes in other components, such as lipids and moisture. In contrast, Xie et al. [42] and Qiu et al. [38] found that body composition remained stable in shrimp-fed plant-based protein mixes, which is consistent with the findings of the present study using CFPA. The stability of proximate components suggests that CFPA is a nutritionally comparable alternative to traditional protein sources in shrimp diets, except for its slight impact on ash content. The rise in ash content may be attributed to the inherent mineral composition of corn coproducts or enhanced mineral retention due to the fermentation process, which could improve mineral bioavailability.

Assessing gene expression in the gut and hepatopancreas of shrimp is essential for elucidating the impact of varying inclusion levels of CFP on health and digestive function. The expression levels of proinflammatory (*tnf-α*), anti-inflammatory (*tgf-β1*), immune-related genes (*propo* and *sod*), and digestive enzymes (*tryp*sin, *amylase*, *alp*, and *fasn*) are critical indicators of shrimp health and digestive response to dietary changes. The gut plays a crucial role in both immune responses and nutrient absorption, with gene expression patterns providing valuable insights into the physiological impacts of dietary interventions [43]. *Tnf-α* is a proinflammatory cytokine that plays a crucial role in initiating and regulating immune responses in shrimp [44, 45]. Upregulation of *tnf-α* expression in the gut of *L. vannamei* fed higher levels of CFPA, suggests that increased CFPA inclusion may induce an inflammatory response in shrimp. This observation is consistent with prior research indicating which indicated increased *tnf-α* expression in shrimp which consumed plant-based diets, including SBM [46] and low FM diets [22], suggesting that plant-derived proteins may influence intestinal inflammation. The lack of substantial alterations in *tgfb1* expression, even with the rising trend associated with higher CFPA inclusion, may indicate an anti-inflammatory regulatory mechanism designed to mitigate inflammation. *Tgfb1* is essential for regulating immune homeostasis by suppressing the excessive inflammatory responses induced by *tnf-α* in shrimp [47, 48]. The simultaneous increase in *tnf-α* and *tgfb1* expression at higher CFPA levels suggests that shrimp may activate regulatory mechanisms to mitigate inflammatory effects induced by plant-based diets. Gene expression patterns suggest that moderate inclusion of CFPA (10%–15%) may enhance immune activation and mitigate chronic inflammation, underscoring the need to optimize CFPA levels in shrimp diets. Investigating the molecular pathways regulating cytokines will enhance the understanding of the immunomodulatory effects of plant-based protein ingredients, thereby promoting the development of sustainable shrimp feed.

The expression of immune-related genes, including *propo* and *sod*, is essential for the innate immunological defense of shrimp [49, 50], especially in response to external stresses such as dietary alterations. *propo* is a precursor enzyme in the *propo* system, which participates in melanization and pathogen identification, constituting the primary immunological response in crustaceans [51, 52]. This study found no significant differences in *propo* expression across dietary treatments; however, shrimp given 10% and 25% CFPA diets showed a propensity for increased *propo* expression relative to the baseline diet. The increase may indicate a slight immune activation due to CFPA inclusion, perhaps signifying the existence of bioactive substances or antinutritional factors in plant-based foods that test the shrimp's immune system. The same trend was noted by Xie et al. [42], who documented elevated *propo* expression in *L. vannamei* when fed diets with a plant protein source used as a replacement for FM, suggesting that plant-derived components may influence shrimp immune responses without substantial inflammatory repercussions. Similarly, the consistent *sod* expression within dietary groups in the current study suggests that oxidative stress levels were steady and indicated no immunological abnormalities. The findings agree with earlier studies that suggested antioxidant enzyme activity can serve as an indicator of shrimp health [23]. Additional examination of other oxidative stress indicators may provide a more comprehensive understanding of the immunomodulatory effects associated with CFPA incorporation in shrimp diets. These findings suggest that CFPA supplementation at higher inclusion levels (25%) may stimulate proinflammatory cytokine expression, potentially indicating an immune-modulatory effect without compromising an antioxidant defense.

The absence of significant changes in the hepatopancreatic gene expression of digestive enzymes (*tryp*sin, *alp*, and *amylase*) in *L. vannamei*-fed CFPA-supplemented diets suggests that CFP isolate serves as a nutritionally sufficient alternative to conventional protein sources, preserving digestive homeostasis without inducing substantial transcriptional responses. Trypsin, a key protease involved in protein digestion [53], showed slight upregulation in the CFPA 10% diet before decreasing at elevated inclusion levels. This trend may indicate the equilibrium between protein availability and protease enzyme regulation, as prior research has demonstrated that high dietary protein or modified protein quality can suppress trypsin gene expression in crustaceans. The downregulation of *alp*, which serves as an indicator of intestinal brush border functionality and nutrient adaptation [54], may suggest decreased digestive activity linked to higher CFPA inclusion. This could be related to changes in protein digestibility or the presence of residual antinutritional factors in CFP products [55]. The sustained downregulation of *amylase* in CFP diets aligns with other studies which have indicated that shrimp modulate carbohydrate digestion in reaction to dietary protein sources [56–58].

Conversely, the nonsignificant upregulated trend in *fasn* expression at higher CFPA inclusions points to a subtle shift in lipid metabolism. *Fasn*, a crucial enzyme in de novo lipogenesis [59], may be upregulated as a compensatory response to changes in AA profiles or energy allocation from CFPA, a phenomenon noted with plant-based proteins in shrimp [60]. The results indicated that the addition of CFPA did not lead to significant changes in the expression of digestive enzyme genes; however, minor transcriptional modifications related to lipid metabolism may occur as part of an adaptive physiological response to the novel protein source. Further research that combines proteomic analysis with the study of nutrient transporters will provide insight into the mechanisms that affect these gene expression patterns.

The results from Trial 2 showed that including CFPB1 or CFPB2 in the diets of juvenile Pacific white shrimp did not significantly enhance growth over the 7-week study. The FCR and survival remained unaffected across dietary treatments, and indicated no discernible impact on feed utilization efficiency, which was similar to the findings of Guo [12] and Nazeer et al. [61]. Moreover, regression analyses revealed distinct responses to CFPB1 and CFPB2 inclusion levels. A quadratic model for CFPB1 suggested a nonlinear relationship between inclusion rate and FW, whereas a linear model for CFPB2 indicated minimal variation across levels. These findings imply differences in nutritional composition and digestibility between CFPB1 and CFPB2. Fermentation may enhance protein quality by reducing antinutritional factors and increasing AA bioavailability, as reported in previous studies [55, 62, 63]. Several studies have reported the successful use of DDGS in shrimp diets [12, 38, 64, 65].

Unlike Trial 1, the diets in Trial 2 were specifically formulated to mirror standard procedures in the Latin American shrimp market, characterized by greater inclusion of animal protein sources, such as FM and PM [66, 67]. This approach aimed to replicate regional dietary compositions and provide practical insights into the application of CFP products in the commercial sector. The diets incorporated a constant quantity of FM (8%) and PM (12%); however, the SBM content decreased as the inclusion levels of CFPB1 or CFPB2 increased to maintain isonitrogenous formulations. This formulation technique ensured that any observed effects were attributed to the replacement of plant protein with CFP rather than a variation in overall protein concentration. Moreover, Trial 2 did not include whole-body composition or gene expression analysis, as Trial 1 results showed no significant differences in growth performance or protein retention across diets with different levels of CFPA. Trial 2 offers insights into the practical viability and commercial application of these alternative ingredients as protein sources in shrimp diets.

## 5. Conclusion

This study illustrated that CFP products, a newly developed ingredient from the ethanol industry produced using advanced processing technology to enhance their nutritional profile compared to traditional ethanol coproducts, effectively replaced SBM in *L. vannamei* diets at 5%–12% inclusion and maintained growth performance, feed efficiency, and proximate composition in a clear water system. Specifically, CFPA at 20% inclusion showed no performance degradation, as well as CFPB1 (8%) and CFPB2 (12%) yielded similar outcomes. Gene expression in the hepatopancreas indicated consistent digestive enzyme activity and immunological equilibrium despite slight inflammatory reactions at elevated inclusion levels. These findings underscore CFP's viability as a sustainable alternative protein source in diets for Pacific white shrimp, with no additional advantages at 20%–25% inclusion. More research into proteome responses and nutrient transport systems is advised to enhance the understanding of CFP application in shrimp aquaculture.

## Figures and Tables

**Table 1:** Proximate composition and amino acid profile of soybean meal and corn-fermented products in the diets for Pacific white shrimp, *Litopenaeus vannamei* in two growth trials.

Proximate^a^ (g 100 g^−1^, as is)	SBM	^b^CFPA	^c^CFPB1	^c^CFPB2
Moisture	13.32	8.30	7.27	6.50
Crude protein	43.28	48.6	50.93	61.16
Crude fat	0.26	4.31	3.61	4.79
Crude fiber	3.65	8.16	7.45	4.71
Ash	5.92	2.18	2.96	2.53
Essential amino acids				
Arginine	3.08	2.09	2.39	2.64
Histidine	1.12	1.34	1.48	1.64
Isoleucine	2.05	2.24	2.31	2.64
Leucine	3.41	6.71	6.78	8.44
Lysine	2.81	1.85	2.01	1.91
Methionine	0.60	0.99	1.25	1.47
Phenylalanine	2.27	2.91	2.99	3.6
Threonine	1.63	1.99	2.14	2.37
Tryptophan	0.55	0.32	0.39	0.43
Valine	2.13	2.77	3.01	3.39
Nonessential amino acids				
Alanine	1.88	3.70	4.00	4.81
Aspartic acid	4.80	3.55	3.82	4.21
Cystine	0.61	0.96	1.04	1.23
Glutamic acid	7.84	8.77	9.25	11.34
Glycine	1.84	1.85	2.16	2.39
Hydroxylysine	0.04	0.08	0.09	0.12
Hydroxyproline	0.11	0.03	0.05	0.05
Lanthionine	0.00	0.00	0.00	0.00
Ornithine	0.04	0.02	0.02	0.03
Proline	2.11	4.16	4.25	5.20
Serine	1.79	2.27	2.45	2.82
Taurine	0.11	0.10	0.09	0.07
Tyrosine	1.67	2.12	2.32	2.84

^a^Analysis conducted by the University of Missouri Agricultural Experimental Station Chemical Laboratories (Columbia, Missouri, USA) (results are expressed on g 100 g^−1^ of feed as is unless otherwise indicated).

^b^Altipro product, Renewable Products Marketing Group, Shakopee, Minnesota, USA.

^c^Ultra high protein products (50% and 60% crude protein), Green Plains, Omaha, Nebraska, USA.

**Table 2:** Formulation of experimental diets (36% protein and 6% lipid on as is basis) to evaluate the effect of corn-fermented protein (CFPA) on growth performance of Pacific white shrimp, *Litopenaeus vannamei* (g 100 g^−1^ as is) in Trial 1.

Ingredients	Basal	CFPA 5%	CFPA 10%	CFPA 15%	CFPA 20%	CFPA 25%
Fish meal^a^	6.00	6.00	6.00	6.00	6.00	6.00
Soybean meal^b^	51.69	48.88	46.08	43.26	40.46	37.89
Corn protein concentrate^c^	8.00	6.40	4.80	3.20	1.60	0.00
Corn-fermented protein A^d^	0.00	5.00	10.00	15.00	20.00	24.80
Fish oil^e^	4.11	3.93	3.75	3.57	3.39	3.22
Lecithin^f^	1.00	1.00	1.00	1.00	1.00	1.00
Cholesterol^g^	0.12	0.12	0.12	0.12	0.12	0.12
Corn starch^g^	3.98	3.57	3.15	2.75	2.33	1.87
Whole wheat^h^	20.00	20.00	20.00	20.00	20.00	20.00
Mineral premix^i^	0.50	0.50	0.50	0.50	0.50	0.50
Vitamin premix^j^	1.80	1.80	1.80	1.80	1.80	1.80
Choline chloride^h^	0.20	0.20	0.20	0.20	0.20	0.20
Stay C^k^	0.10	0.10	0.10	0.10	0.10	0.10
CaP-dibasic^l^	2.50	2.50	2.50	2.50	2.50	2.50
Proximate composition^m^ (g 100 g^−1^, as is)						
Moisture	7.81	7.67	6.93	6.50	9.01	7.07
Crude protein	38.56	38.35	39.11	38.74	37.87	38.68
Crude fat	6.87	6.68	6.51	6.73	6.52	6.77
Crude fiber	3.80	3.98	4.80	4.77	5.27	5.56
Ash	6.77	6.80	6.74	6.58	6.34	6.35

^a^Omega Protein Inc, Houston, Texas, USA.

^b^Solvent extracted soybean meal, Auburn University, Auburn, Alabama, USA.

^c^Empyreal 75 TM cargill corn milling, Cargill Inc., Blair, Nebraska, USA.

^d^Altipro product, Renewable Products Marketing Group, Shakopee, Minnesota, USA.

^e^Omega Protein Inc., Reedville, Virginia, USA.

^f^The Solae Company, St. Louis, Missouri, USA.

^g^MP Biomedicals Inc., Solon, Ohio, USA.

^h^Bobs Red Mill, Milwaukie, Oregon, USA.

^i^Trace mineral premix (g 100 g^−1^premix): cobalt chloride, 0.004; cupric sulfate pentahydrate, 0.550; ferrous sulfate, 2.000; magnesium sulfate anhydrous, 13.862; manganese sulfate monohydrate, 0.650; potassium iodide, 0.067; sodium selenite, 0.010; zinc sulfate heptahydrate, 13.193; alpha-cellulose, 69.664.

^j^Vitamin premix (g kg^−1^ premix): thiamin HCL, 4.95; riboflavin, 3.83; pyridoxine HCL, 4.00; Ca-pantothenate, 10.00; nicotinic acid, 10.00; biotin, 0.50; folic acid, 4.00; cyanocobalamin, 0.05; inositol, 25.00; vitamin A acetate (500,000 IU g^−1^), 0.32; vitamin D3 (1,000,000 IU g^−1^), 80.00; menadione, 0.50; alpha-cellulose, 856.81.

^k^Stay C, (L-ascorbyl-2-polyphosphate 35% Active C), Roche Vitamins Inc., Parsippany, New Jersey, USA.

^l^VWR Amresco, Suwanee, Georgia, USA.

^m^Analysis conducted by the University of Missouri Agricultural Experimental Station Chemical Laboratories (Columbia, Missouri, USA) (results are expressed on g 100 g^−1^ of feed as is, unless otherwise indicated).

**Table 3:** Formulation of experimental diets (36% protein and 6% lipid on as is basis) to evaluate the effect of corn-fermented protein (CFPB1/2) on growth performance of Pacific white shrimp, *Litopenaeus vannamei* (g 100 g^−1^ as is) in Trial 2.

Ingredients	Basal	CFPB1 4%	CFPB1 8%	CFPB1 12%	CFPB1 16%	CFPB2 8%	CFPB2 12%	CFPB2 16%
Fish meal^a^	8.00	8.00	8.00	8.00	8.00	8.00	8.00	8.00
Poultry meal^b^	12.0	12.00	12.00	12.00	12.00	12.00	12.00	12.00
CFPB2^c^	0.00	0.00	0.00	0.00	0.00	8.00	12.00	16.00
CFPB1^c^	0.00	4.00	8.00	12.00	16.00	0.00	0.00	0.00
Soybean meal^d^	42.6	38.00	33.40	28.70	24.10	31.70	26.20	20.80
Fish oil^e^	2.90	2.76	2.63	2.49	2.35	2.66	2.54	2.42
Lecithin^f^	1.00	1.00	1.00	1.00	1.00	1.00	1.00	1.00
Cholesterol^g^	0.10	0.10	0.10	0.10	0.10	0.10	0.10	0.10
Corn Starch^g^	1.30	2.04	2.77	3.61	4.35	4.44	6.06	7.58
Whole wheat^h^	27.0	27.00	27.00	27.00	27.00	27.00	27.00	27.00
Mineral premix^i^	0.50	0.50	0.50	0.50	0.50	0.50	0.50	0.50
Vitamin premix^j^	1.80	1.80	1.80	1.80	1.80	1.80	1.80	1.80
Choline chloride^h^	0.20	0.20	0.20	0.20	0.20	0.20	0.20	0.20
Stay-C active 35%^k^	0.10	0.10	0.10	0.10	0.10	0.10	0.10	0.10
CaP-dibasic^l^	2.50	2.50	2.50	2.50	2.50	2.50	2.50	2.50
^m^Proximate composition (g 100 g^−1^, as is)								
Crude protein	37.48	37.38	37.02	36.82	37.22	36.89	37.15	36.85
Moisture	9.43	9.39	9.07	9.40	8.22	9.80	9.04	8.80
Crude fat	6.86	6.71	6.72	6.72	6.54	6.63	6.78	7.60
Crude fiber	3.43	3.49	3.82	3.74	3.80	3.43	3.53	3.13
Ash	8.11	7.96	7.67	7.46	7.53	7.58	7.56	7.14

^a^Omega Protein Inc, Houston, Texas, USA.

^b^Poultry meal, Auburn University, Auburn, Alabama, USA.

^c^Corn-fermented protein products, Green Plains Inc, Omaha, Nebraska, USA.

^d^Solvent extracted soybean meal, Auburn University, Auburn, Alabama, USA.

^e^Omega Protein Inc., Reedville, Virginia, USA.

^f^The Solae Company, St. Louis, Missouri, USA.

^g^MP Biomedicals Inc., Solon, Ohio, USA.

^h^Bobs Red Mill, Milwaukie, Oregon, USA.

^i^Trace mineral premix (g 100 g^−1^ premix): cobalt chloride, 0.004; cupric sulfate pentahydrate, 0.550; ferrous sulfate, 2.000; magnesium sulfate anhydrous, 13.862; manganese sulfate monohydrate, 0.650; potassium iodide, 0.067; sodium selenite, 0.010; zinc sulfate heptahydrate, 13.193; alpha-cellulose, 69.664.

^j^Vitamin premix (g kg^−1^ premix): thiamin HCL, 4.95; riboflavin, 3.83; pyridoxine HCL, 4.00; Ca-pantothenate, 10.00; nicotinic acid, 10.00; biotin, 0.50; folic acid, 4.00; cyanocobalamin, 0.05; inositol, 25.00; vitamin A acetate (500,000 IU g^−1^), 0.32; vitamin D3 (1,000,000 IU g^−1^), 80.00; menadione, 0.50; alpha-cellulose, 856.81.

^k^Stay C, (L-ascorbyl-2-polyphosphate 35% Active C), Roche Vitamins Inc., Parsippany, New Jersey, USA.

^l^VWR Amresco, Suwanee, Georgia, USA.

^m^Analysis conducted by the University of Missouri Agricultural Experimental Station Chemical Laboratories (Columbia, Missouri, USA).

**Table 4:** Amino acid profile (g 100 g^−1^, as is) of test diets (36% protein and 6% lipid on as is basis) fed to Pacific white shrimp, *Litopenaeus vannamei* in Trial 1.

Amino acids	Basal	CFPA 5%	CFPA 10%	CFPA 15%	CFPA 20%	CFPA 25%
Essential amino acids						
Arginine	2.37	2.35	2.33	2.30	2.21	2.26
Histidine	1.00	1.00	1.02	1.03	1.02	1.06
Isoleucine	1.73	1.70	1.69	1.80	1.75	1.80
Leucine	3.56	3.53	3.56	3.54	3.56	3.62
Lysine	2.09	2.07	2.08	2.10	2.01	2.10
Methionine	0.65	0.64	0.66	0.66	0.64	0.67
Phenylalanine	2.01	1.99	2.00	1.97	1.94	1.99
Threonine	1.42	1.43	1.45	1.38	1.37	1.41
Tryptophan	0.43	0.43	0.43	0.43	0.40	0.41
Valine	1.84	1.83	1.84	1.98	1.95	2.02
Nonessential amino acids						
Alanine	2.03	2.04	2.08	2.07	2.10	2.13
Aspartic acid	3.66	3.62	3.59	3.57	3.44	3.54
Cysteine	0.56	0.60	0.60	0.62	0.61	0.64
Glutamic acid	7.45	7.36	7.31	7.27	7.08	7.18
Glycine	1.65	1.66	1.69	1.70	1.67	1.72
Hydroxylysine	0.00	0.00	0.00	0.01	0.01	0.01
Hydroxyproline	0.12	0.14	0.14	0.13	0.09	0.14
Lanthionine	0.11	0.11	0.13	0.12	0.15	0.15
Ornithine	0.06	0.06	0.06	0.06	0.06	0.06
Proline	2.35	2.38	2.43	2.42	2.45	2.52
Serine	1.65	1.66	1.69	1.50	1.47	1.52
Taurine	0.24	0.24	0.25	0.23	0.23	0.25
Tyrosine	1.45	1.44	1.44	1.38	1.37	1.37
Sum of amino acids	38.43	38.28	38.47	38.27	37.58	38.57

*Note:* Analysis was conducted by the University of Missouri Agricultural Experimental Station Chemical Laboratories (Columbia, Missouri, USA).

**Table 5:** Amino acid profile (g 100 g^−1^, as is) of test diets fed to Pacific white shrimp, *Litopenaeus vannamei* in Trial 2.

Amino acid^a^	Basal	CFPB1 4%	CFPB1 8%	CFPB1 12%	CFPB1 16%	CFPB2 8%	CFPB2 12%	CFPB2 16%
Essential amino acids								
Arginine	2.49	2.29	2.29	2.18	2.21	2.21	2.14	2.03
Histidine	0.94	0.90	0.92	0.91	0.93	0.90	0.89	0.89
Isoleucine	1.68	1.60	1.62	1.58	1.60	1.57	1.54	1.57
Leucine	2.81	2.79	2.92	2.98	3.16	2.95	3.08	3.25
Lysine	2.31	2.12	2.14	2.05	2.02	2.06	1.96	1.85
Methionine	0.67	0.67	0.71	0.69	0.74	0.70	0.71	0.74
Phenylalanine	1.79	1.71	1.74	1.72	1.78	1.73	1.73	1.74
Threonine	1.44	1.37	1.40	1.38	1.42	1.38	1.37	1.33
Tryptophan	0.46	0.43	0.43	0.40	0.40	0.41	0.41	0.37
Valine	1.86	1.84	1.88	1.85	1.90	1.82	1.82	1.88
Nonessential amino acids								
Alanine	1.87	1.87	1.98	2.00	2.11	1.98	2.07	2.15
Aspartic acid	3.67	3.39	3.39	3.25	3.22	3.30	3.15	3.02
Cysteine	0.53	0.52	0.55	0.54	0.57	0.54	0.52	0.55
Glutamic acid	6.84	6.51	6.65	6.55	6.64	6.63	6.52	6.54
Glycine	2.13	2.07	2.16	2.05	2.08	2.07	2.08	2.05
Hydroxylysine	0.08	0.08	0.08	0.08	0.08	0.08	0.09	0.08
Hydroxyproline	0.32	0.33	0.34	0.33	0.30	0.32	0.33	0.34
Lanthionine	0.01	0.00	0.00	0.00	0.00	0.00	0.00	0.00
Ornithine	0.03	0.03	0.03	0.02	0.03	0.03	0.03	0.03
Proline	2.21	2.21	2.32	2.34	2.46	2.32	2.40	2.49
Serine	1.53	1.45	1.50	1.49	1.52	1.50	1.47	1.41
Taurine	0.26	0.27	0.27	0.28	0.28	0.27	0.29	0.28
Tyrosine	1.25	1.14	1.20	1.20	1.26	1.16	1.18	1.14
Sum of amino acids	37.18	35.59	36.52	35.87	36.71	35.93	35.78	35.73

*Note:* Diets were formulated with 36% protein and 6% lipid on as is basis.

^a^Analysis was conducted by University of Missouri Agricultural Experimental Station Chemical Laboratories (Columbia, Missouri, USA).

**Table 6:** Primers used for real-time qPCR analysis to assess physiological and digestive gene expression of Pacific white shrimp, *Litopenaeus vannamei* cultured in clear water recirculating systems for 6 weeks, fed corn-fermented protein (CFPA) at various levels with an initial weight of 1.02 ± 0.02 g (mean ± standard deviation).

Gene	Forward primer (5′–3′)	Reverse primer (5′–3′)	Efficiency (%)	References
Cytokines				
* tnf-α*	CTCAGCCATCTCCTTCTTG	TGTTCTCCTCGTTCTTCAC	102.7	[21]
* tgf-β1*	AACCATGCCCTTGTGCAAAC	CTTTGGGGGAACCTCGGTC	104.3	[22]
Immunes				
* propo*	TACATGCACCAGCAAATTATCG	AGTTTGGGGAAGTAGCCGTC	107.7	[23]
* sod*	GCAATGAATGCCCTTCTACC	CAGAGCCTTTCACTCCAACG	101.6	[24]
Digestive				
* tryp*sin	TCCAAGATCATCCAACACGA	GACCCTGAGCGGGAATATC	105.2	[25]
* amylase*	CATCTCCGGGTCGAAGGACG	GGCGTCCGACACCTTACAGT	99.82	Genbank (XM070144253.1)
* alp*	CAGCTGGTCTACGGGGCTAC	CTTGCCGTCGTCACGCTTAC	100.53	Genbank (XM070130392.1)
* fasn*	GTCCTCACCTCACGAAGGGG	CAGCGCCCTCAGGAGTAGAC	96.98	Genbank (XM070128169.1)
Reference gene				
* l21*	GTTGACTTGAAGGGCAAT G	CTTCTTGGCTTCGATTCTG	98.6	[26]
* ef1α*	GTATTGGAACAGTGCCCGTG	TCACCAGGGACAGCCTCAGTA	100.3	[27]

Abbreviations: *alp*, alkaline phosphatase; *fasn*, fatty acid synthase; *propo*, prophenoloxidase; *sod*, superoxide dismutase; *tgf-β1*, transforming growth factor beta-1; *tnf-α*, tumor necrosis factor-alpha.

**Table 7:** Summary of water quality parameters (mean ± SD) recorded during the trials conducted to evaluate growth in Trial 1 and Trial 2 of juvenile Pacific white shrimp, *Litopenaeus vannamei* fed different corn-fermented protein (CFP) products levels.

Parameter	Trial 1	Trial 2
Dissolved oxygen (mg/L)	7.23 ± 0.32	6.95 ± 0.27
Temperature (°C)	28.07 ± 0.35	27.76 ± 0.17
Salinity (g/L)	9.78 ± 0.24	9.49 ± 0.22
pH	8.02 ± 0.15	8.05 ± 0.15
Total ammonia nitrogen (mg/L)	0.08 ± 0.05	0.12 ± 0.07
Nitrite nitrogen (mg/L)	0.14 ± 0.14	0.23 ± 0.27

**Table 8:** Performance of juvenile Pacific white shrimp, *Litopenaeus vannamei* (mean initial weight 1.02 ± 0.02 g) fed diets (36% protein and 6% lipid) containing varying corn-fermented protein levels (CFPA) within a 6-week period in Trial 1.

Parameters	Final weight (g)	Weight gain (g)	Weight gain (%)	TGC	FCR	Survival (%)	ANPR (%)
Basal	10.49	9.48	933	0.27	1.46^b^	92.22	35.51
^1^CFPA 5%	10.78	9.77	962	0.28	1.46^b^	87.78	35.72
CFPA 10%	10.49	9.47	933	0.27	1.55^ab^	85.56	33.79
CFPA 15%	10.03	9.02	888	0.26	1.50^ab^	93.33	32.73
CFPA 20%	10.47	9.45	932	0.27	1.61^a^	82.22	34.64
CFPA 25%	10.19	9.17	903	0.26	1.54^ab^	90.00	32.75
PSE	0.18	0.18	19	0.01	0.03	3.11	0.85
*p*-Value	0.095	0.093	0.124	0.048	0.007	0.140	0.062
Regression							
*R*^2^	0.092	0.091	0.081	0.099	0.218	0.015	0.153
*p*-Value	0.072	0.075	0.093	0.061	0.004	0.473	0.018

*Note:* Values represent means of six replicates. Means not sharing any lowercase superscript letters are significantly different by the Tukey's HSD test at the 5% level of significance.

Abbreviations: ANPR, apparent net protein retention; FCR, Feed conversion ratio; PSE, Pooled standard error; TGC, Thermal-unit growth coefficient.

^1^CFPA, corn-fermented protein (Altipro product, RPMG, Shakopee, Minnesota, USA).

**Table 9:** Whole-body (g 100 g^−1^ dry weight) proximate composition of Pacific white shrimp, *Litopenaeus vannamei* (initial mean weight 1.02 ± 0.02 g) cultured in a clear water recirculating system for 6 weeks fed diets (36% protein and 6% lipid) containing varying corn-fermented protein levels (CFPA) in Trial 1.

Proximate analysis	Moisture^a^ (%)	Dry matter (%)	Crude protein (%)	Crude fat (%)	Crude fiber (%)	Ash (%)
Basal	76.78	23.22	76.28	6.85	7.95	11.4
CFPA 5%	77.08	22.92	76.3	6.55	5.56	11.07
CFPA 10%	77.13	22.87	76.77	6.79	6.5	11.05
CFPA 15%	77.43	22.57	76.77	5.8	6.94	11.83
CFPA 20%	76.96	23.04	76.03	6.35	6.36	11.85
CFPA 25%	77.77	22.23	76.97	6.38	6.13	11.95
PSE	0.42	0.42	0.43	0.34	0.69	0.26
*p*-Value	0.615	0.615	0.614	0.303	0.259	0.05
Regression						
*R*^2^	0.059	0.059	0.016	0.054	0.032	0.177
*p*-Value	0.154	0.154	0.465	0.173	0.298	0.011

*Note:* Proximate analysis performed by Midwest Laboratories (Omaha, Nebraska, USA). Values represent the mean of six replicates of each diet (*n* = 4).

Abbreviation: PSE, pooled standard error.

^a^Based on an as-is basis. CFPA, corn-fermented protein A (Altipro product, RPMG, Shakopee, Minnesota, USA).

**Table 10:** Response of juvenile Pacific white shrimp, *Litopenaeus vannamei* (initial mean weight 0.55 ± 0.012 g) fed diets containing graded levels (0%, 4%, 8%, 12%, and 16%) of corn-fermented products (CFPB1/2) over a 7-week period in Trial 2.

Diets	Final weight (g)	Weight gain (g)	Weight gain (%)	TGC	FCR	Survival (%)	Weekly weight gain (g)
Basal	12.34	11.78	2120	0.29	1.38	89.33	1.68
^a^CFPB1 4%	12.25	11.69	2096	0.28	1.47	88.00	1.67
CFPB1 8%	12.67	12.12	2174	0.29	1.40	86.67	1.73
CFPB1 12%	12.48	11.92	2136	0.29	1.44	86.67	1.70
CFPB1 16%	11.99	11.43	2055	0.28	1.52	84.00	1.63
PSE	0.20	0.21	44	0.01	0.07	4.28	0.03
*p*-Value	0.221	0.225	0.418	0.381	0.591	0.927	0.194
Basal	12.34	11.78	2120	0.29	1.38	89.33	1.68
^a^CFPB2 8%	12.55	11.99	2151	0.29	1.42	88.00	1.71
CFPB2 12%	12.60	12.05	2165	0.29	1.45	84.00	1.72
CFPB2 16%	12.26	11.70	2105	0.28	1.42	90.67	1.67
PSE	0.25	0.25	52	0.01	0.04	3.53	0.04
*p*-Value	0.759	0.723	0.831	0.701	0.773	0.586	0.699
ANCOVA							
Ingredient	0.575	0.580	0.654	0.414	0.941	0.569	0.542
Level	0.206	0.212	0.334	0.214	0.637	0.968	0.194
Ingredient × level	0.790	0.798	0.861	0.591	0.749	0.453	0.770

*Note:* Values represent means of five replicates. Means not sharing any letters are significantly different by the Tukey's HSD-test at the 5% level of significance.

Abbreviations: FCR, feed conversion ratio; PSE, pooled standard error; TGC, thermal-unit growth coefficient.

^a^CFPB, corn-fermented protein B1 (50% crude protein) and B2 (60% protein) (Green Plains Inc, Omaha, Nebraska, USA).

## Data Availability

The data is available upon request from the authors.
